# SNPranker 2.0: a gene-centric data mining tool for diseases associated SNP prioritization in GWAS

**DOI:** 10.1186/1471-2105-14-S1-S9

**Published:** 2013-01-14

**Authors:** Ivan Merelli, Andrea Calabria, Paolo Cozzi, Federica Viti, Ettore Mosca, Luciano Milanesi

**Affiliations:** 1 Consiglio Nazionale delle Ricerche - Istituto di Tecnologie Biomediche (CNR-ITB), Via F.lli Cervi 93, 20090 Segrate (MI), Italy; 2 San Raffaele Telethon Institute for Gene Therapy (HSR-TIGET), Via Olgettina 58, 20132 Milano, Italy; 3 Parco Tecnologico Padano, Via Einstein - Loc. Cascina Codazza, 26900 Lodi, Italy

## Abstract

**Background:**

The capability of correlating specific genotypes with human diseases is a complex issue in spite of all advantages arisen from high-throughput technologies, such as Genome Wide Association Studies (GWAS). New tools for genetic variants interpretation and for Single Nucleotide Polymorphisms (SNPs) prioritization are actually needed. Given a list of the most relevant SNPs statistically associated to a specific pathology as result of a genotype study, a critical issue is the identification of genes that are effectively related to the disease by re-scoring the importance of the identified genetic variations. Vice versa, given a list of genes, it can be of great importance to predict which SNPs can be involved in the onset of a particular disease, in order to focus the research on their effects.

**Results:**

We propose a new bioinformatics approach to support biological data mining in the analysis and interpretation of SNPs associated to pathologies. This system can be employed to design custom genotyping chips for disease-oriented studies and to re-score GWAS results. The proposed method relies (1) on the data integration of public resources using a *gene-centric *database design, (2) on the evaluation of a set of static biomolecular annotations, defined as *features*, and (3) on the SNP scoring function, which computes SNP scores using parameters and weights set by users. We employed a machine learning classifier to set default feature weights and an ontological annotation layer to enable the enrichment of the input gene set. We implemented our method as a web tool called *SNPranker 2.0 *(http://www.itb.cnr.it/snpranker), improving our first published release of this system. A user-friendly interface allows the input of a list of genes, SNPs or a biological process, and to customize the features set with relative weights. As result, SNPranker 2.0 returns a list of SNPs, localized within input and ontologically enriched genes, combined with their prioritization scores.

**Conclusions:**

Different databases and resources are already available for SNPs annotation, but they do not prioritize or re-score SNPs relying on a-priori biomolecular knowledge. SNPranker 2.0 attempts to fill this gap through a user-friendly integrated web resource. End users, such as researchers in medical genetics and epidemiology, may find in SNPranker 2.0 a new tool for data mining and interpretation able to support SNPs analysis. Possible scenarios are GWAS data re-scoring, SNPs selection for custom genotyping arrays and SNPs/diseases association studies.

## Background

The increasing importance of high throughput molecular biology techniques, such as whole genome genotyping and next generation sequencing, has boosted the identification of novel biomarkers for diseases having a genetic component [1,2]. In particular, the evaluation of Single Nucleotide Polymorphisms (SNPs) is very promising, because they represent established single base variations with respect to the wild type, and their knowledge can be exploited to characterize each subject by associating a specific phenotype with the corresponding genomic pattern.

The human genome counts more than 10 million of SNPs [3] and the number of SNPs with a minor allele frequency over 10% is estimated to be perhaps as many as five million [4]. SNPs are distributed throughout the human genome and their effect on phenotype depends on the biological role (e.g. exon or regulatory) and state (e.g. silent or active) of the genomic regions where they occur.

SNP knowledge is widely exploited for Genome Wide Association Studies (GWAS) [5-7], identification of Copy Number Variations (CNV) [8], and observations about Population Stratification [9]. Nowadays, chip technologies allow the analysis of up to one million SNPs for each patient. The selection of SNPs to be included in the analysis is a critical problem for genotype array providers, which employ the non-random inheritance of these genomic variations to identify TAG SNPs representing haplotype blocks. A widely used approach to optimize the SNP probe set relies on the concept of Linkage Disequilibrium (LD) [10], which exploits a statistical similarity measure between adjacent SNPs to compute, for each couple of SNPs, the information improvement using both of them or only the most representative one. LD mapping is used to optimize the experimental information content by containing the number of probes employed for the genotype analysis into 1 million of TAG SNPs.

SNPs filtering and prioritizing methods are also very important in case of custom genotyping chip design, defining disease-oriented arrays by pre-selecting a set of SNPs that can be related to a specific pathology. In this general scenario, no automatic methods have been proposed to support the identification of the most probable SNPs associated to a pathology relying on the available biomolecular knowledge.

On the other hand, GWAS can identify SNPs associated to a disease working on genotypes and phenotypes analysis. Generally, a GWAS output has to be interpreted considering the biological context to enrich the pure statistical results, in which the effective disease related variations could be dispersed among many less critical SNPs. This process means to "re-rank" GWAS scores relying on SNP properties (annotations), in order to shed light on variations that are effectively critical for the pathology in analysis.

Herein we describe SNPranker 2.0, a system that enables the prioritization of SNPs, which relies on a previous published version of the system [11]. SNPranker 2.0 ranks SNPs according to a user-selected set of features, which in this version has been enriched with epigenetics and functional genomics attributes, by employing a novel data mining approach. SNPranker 2.0 provides a machine learning derived scoring schema, which consists of a data mining model, optimized against experimental evidences by employing a genetic algorithm, for characterizing SNPs related to an input dataset of genes, biological processes or GWAS results. The system provides a ranked list of SNPs as output, with annotations about their statistical enrichment with respect to the most represented pathologies.

### Related works

The bioinformatics analysis of genotype experiments is a complex task, which is usually addressed with statistical methods, if sufficient knowledge is available to formulate hypotheses, or using machine learning approaches, if it is necessary to create classificatory rules relying on data themselves.

Statistical approaches are commonly used in genetic epidemiology and in many researches these methods achieved good results [12,13]. Despite these successes, they show some limits, which are mainly related to the underlying statistical hypotheses. The computation of *P*-values, which is a typical approach in GWAS, is prone to bias in the selection of the studied population and the capability of inferring correlations between genomic variations and pathologies is inevitably restricted to the set of TAG SNPs used as probe set (although a posteriori imputing techniques can partially correct this issue, at the price of a huge amount of computation). Statistical approaches are solid, but the abstraction they use to manage data often provides results difficult to interpret, because best hits are selected without any correlation to real genomic features that can be identified as causes of the disease.

On the other hand, machine learning approaches are very flexible thanks to their ability to directly create a model from the data, although in well defined analysis context (i.e. when hypotheses of statistical methods are very solid) are considered less reliable. Considering SNP prioritization as a classification problem, we chose a supervised machine learning approach to generate a function able to map inputs to desired outputs. While employing a supervised method, the selection of the training and validation sets must be carefully achieved, usually employing a cross-validation approach, since the model is created on them.

Machine learning approaches have a long tradition in bioinformatics, which requires the development of tools and methods capable of transforming 'omics' data into real knowledge about the biological underlying mechanism [14-16]. Nonetheless, there are only few applications developed to exploit machine learning approaches for genetic features ranking in relation to specific diseases. An example is Endeavour [17], a software that performs gene prioritization for ranking candidate genes involved in biological processes or diseases relying on their similarity to known genes related to these phenomena.

Concerning SNP prioritization some solutions are available, such as PupaSNP Finder [18], Wjst's system [19], PolyMAPr [20] and SNPselector [21]. These servers usually integrate information from a variety of databases and analytical tools in order to create a knowledge base for SNP annotation, starting from public domain databases, such as dbSNP [22], GoldenPath [23] and SNPper [24], which contain well-organized catalogues of SNPs and provide portals to search for fundamental information about them. More recent solutions, which can be used in the frame of GWAS, are FastSNP [25], which employs a complete decision tree to assign risk rankings for SNP prioritization, F-SNP [26] that integrates more than 16 features for SNP annotation, SPOT [27] that relies on GIN (Genomic Information Networks) scores which are cumulative measures of the biological relevance obtained by combining information across multiple domains, and FitSNP [28] that provides predictions about SNPs involved in diseases relying on a meta-analysis of microarray data. Even an R package available in Bioconductor [29] has been developed in this context, based on variance prioritization, which selects SNPs having significant heterogeneity in variance per genotype using a pre-determined *P*-value threshold.

The first version of SNPranker [11] was also a web tool for SNP prioritization. As many of the listed resources, it relied on a data-warehouse approach for collecting as many data about SNP features as possible, to provide users the most complete annotation schema according to the public available information. The innovation of SNPranker concerned the use of an ontological expansion to enrich the set of input SNPs with data about semantic-associated genomic traits that could have statistical correlations and functional influences on the data provided by users. Nonetheless, in the first version of SNPranker, as in many of the discussed solutions, users must select weights of the SNP features upon their expertise. At the best of our knowledge, no methods are available in literature to evaluate SNPs by features scoring through machine learning algorithm using data mining approaches.

## Methods

**Figure 1:**
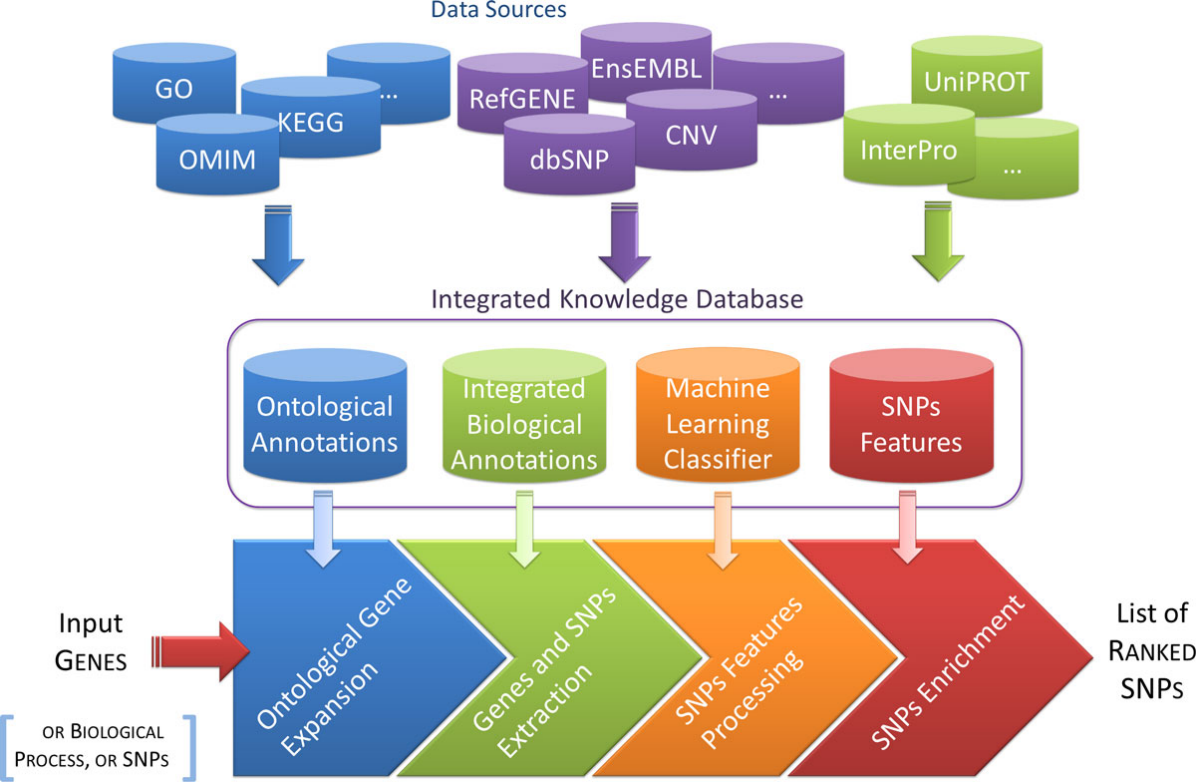
**General schema of SNPranker 2.0 pipeline**. The SNPranker 2.0 reference database collects data from various public data sources using a gene-centric design. This infrastructure represents the core of the SNPranker 2.0 pipeline, which starts with a list of genes (or a biological process or a list of SNPs) as input. If required, the ontological expansion retrieves all genes related to input ones according to a user defined similarity measure and threshold. SNPranker 2.0 performs the score computation using selected features and their corresponding weights, and a final table of SNPs is returned to users with a prioritization score for each SNP.

The core of the designed system can be decoupled into five levels: (1) *data integration*, which consists in the creation of an integrated database starting from sparse and heterogeneous biomolecular annotation data sources; (2) *ontological expansion*, which enables the exploitation of gene ontological annotations to enrich the initial list of genes provided as input; (3) *features set definition*, which includes the choice of the features characterizing each SNP and the related weight in the scoring computation; (4) *scoring computation *through web interface, which is the computation of the function that provides a final score for each SNP; (5) *dataset enrichment analysis*, which allows to verify the statistical significance of most represented diseases and pathways. Figure 1 describes the logical schema of the SNPranker 2.0 pipeline. Following subsections present in details the levels mentioned above.

### Data integration

Similarly to the first implementation of SNPranker, SNPranker 2.0 relies on a data-warehouse architecture, which integrates public information about genes and genes products, in order to provide a solid knowledge base for the SNP scoring engine. As discussed in our previous work [11], the advantage of this database is the use of a strong systems biology approach for data organization, combined with an ontology layer for the annotation of retrieved data. An improvement of SNPranker 2.0 is the use of the NDB engine of MySQL Cluster as backend server that, in combination to the optimization of the database schema, overcomes the latency problem of some complex query requests.

The peculiarity of the developed database is represented by the multi-level approach to data integration [30], which enables a more comprehensive view of the examined process or disease, therefore leading to a better selection of the set of SNPs to be included in a disease-oriented custom chip or a better re-scoring of GWAS data.

The SNPranker 2.0 database presents a *gene-centric *approach, which means that all tables are related to each other using the concept of gene to create relation in the data-warehouse schema, allowing the connection of molecular levels to the pathway level. Human genes are annotated employing, among other features, their symbols, descriptions, aliases and sequences. Data about SNPs are downloaded from GoldenPath [23], with reference to the hg18 genome assembly, which allows the integration of data about chromosomal and contig positions, heterozygosity, alleles and functions of the related DNA portions. Data about known genes and SNPs involvement in particular diseases have been downloaded from OMIM [31].

From the epigenetics point of view, UCSC tracks about DNAse clusters, chromatin structures and methylation patterns have been downloaded and integrated in our database for different tissues and cell lines in order to characterize the specific activity of SNPs in particular environments.

Concerning transcriptomics data, gene products have been collected as lists of mRNA sequences, considering alternative splicing patterns and miRNA binding regions [32], which can be useful to characterize SNPs in the corresponding DNA regions. Since SNPs can modify the mRNA produced from the same locus, by varying the transcription start sites (TSSs), the protein coding DNA sequences (CDSs) or the untranslated regions (UTRs), gene isoforms are also stored in the SNPranker 2.0 database, according to the NCBI RefSeq annotations [33].

Data concerning proteins have been retrieved from UNIPROT [34], for the identification of functional domains, and from the Protein Data Bank [35], to integrate information about structural models.

The systems biology knowledge base has been created by querying databases of biochemical pathways (KEGG [36]) and reactions (Reactome [37]) searching all human gene products, while information about protein-protein interactions (PPIs), collected from BioGRID [38], have been employed to complement the available data about hub proteins and neighbourhoods that are crucial for network based analyses.

SNPranker 2.0 exploits this multilevel knowledge integration as key infrastructure to perform SNP scoring. In this updated version of the database the set of features considered for SNP prioritization consists of more than 30 elements. A complete overview of integrated features is presented in Additional File 1.

### Ontological expansion

The SNPranker 2.0 database has been built on a strong ontology layer, in order to provide a reliable framework for data integration and an improved engine for gene and SNP lists enrichment and annotation. In particular, genes and pathways data have been annotated with terms from the Gene Ontology [39] and the KEGG Pathway Ontology, respectively. By exploiting the ontological annotation of genes, in fact, it is possible to measure gene similarity, which then can be used to expand the initial gene lists. SNPranker 2.0 provides two similarity measures, which differ for taking into account the bare ontological terms (Rel measure [40-42]) or for considering also the ancestor terms following the ontological tree (Wang measure [43]).

Considering one of the ontologies provided by SNPranker 2.0 and a particular similarity measure, from an input gene list *g_1 _*the system generates the list *g_2 _**g_1_*, which contains also the genes that correlate with the genes in the list g_1 _according to the selected similarity threshold.

### Features weight definition

The *features *are the characteristics of SNPs that represent the *a priori *knowledge of their underlying biology, which is the base for modelling the biomolecular information related to these polymorphisms. In this data mining approach, users can select the features they would like to consider for SNP evaluation and assign a custom relevance to these features in the score computation.

An added value of this work is the pre-computation of an optimal set of weights for the features to provide by default suitable ranked SNP lists associated to the disease genes provided as input. The idea is to give a general scoring model, which can predict the importance of each attribute in a generic pathological context, assuring a valuable SNP ranking. A machine learning approach has been used to find this parameter setting, which is proposed by default in the SNPranker 2.0 web site. To find the appropriate weights, we formulated an optimization problem, solved using a genetic algorithm, which considers as fitness the system sensitivity and involves cross-validation during the assessment of candidate weights. In other words, we exploit a genetic algorithm to optimize a model from the data, which is a classical method of supervised machine learning. The combination of this machine learning approach with a framework that allows users to perform a fine-tuning of the system parameters (useful for verifying the effect of changes in the features and relative weights on the final SNP scores) realizes the data mining approach.

In detail, feature weights must be processed through the scoring function in order to obtain a single and significant score for each SNP. The scoring function g  maps the values returned by the selected features and the weights vector $w\in {\mathbb{R}}^{n}$ (where *n *is the number of features) to a single final value, used to calculate the SNP ranking. The function g  is computed as the sum of the values returned by the single features according to their weights w :

(1) $$g:\left(\ldots f_{n},w\right)\mapsto w_{1}f_{1}+w_{2}f_{2}+\ldots +w_{n}f_{n}$$

This strategy allows the computation of the final SNP score as a single real number.

Starting from a set of genes associated by experimental evidence to specific pathologies, a genetic algorithm has been implemented in order to achieve the w  that minimizes the distance among the set of SNPs retrieved by the system and the list of SNPs experimentally associated to the same disease. The optimal values of the weights w  were found taking into account the specificity of the SNPranker 2.0 predictions. To this end, we considered, as input, all the genes and SNPs associated with a set of 16 pathologies, reported in Additional File 2, as described in OMIM. Given the set *S *of SNPs $s_{i}\left(i\in \left\{1,2,...,\left|S\right|\right\}\right)$ containing all the SNPs associated to the genes of the considered pathologies, taking into account a flanking region of 100,000 bp, and defining *A *as the set of SNPs certified by OMIM as involved in the disease, we minimise

(2) $${\text{argmin}}_{w\in {\mathbb{R}}^{30}}\left(1-\frac{\left|P\right|}{\left|P|+|N\right|}\right)$$

where the sets P⊆A is the set of true positives included in the high scoring SNP list. $Y=\text{\{}s_{i}|\sigma_{i}\left(w\right)>\varepsilon \}$, where $\sigma_{i}\left(w\right)$ is the score of the SNP $s_{i}$ and ε  is a constant value and $N\subseteq A\left(P\cap N=\emptyset \right)$ is the false set of negatives. The optimisation process was carried out by evaluating feature coefficients by employing a genetic algorithm for all the pathologies in our set. The system was implemented in Python and the machine learning approach was developed employing the Pygene library [44]. Score calculations and SNP sorting were implemented by embedding C code with SWIG [45], in order to minimize the time needed for fitness evaluations. In our model, all individuals are generated randomly by selecting coefficients between 0 and 1. The fitness is calculated first by filtering scores with a threshold $T_{\varepsilon }$ defined as

(3) $$T_{\varepsilon }=\varepsilon \sigma_{i}\left(w\right)$$

where ε  is computed with steps of 0.1 starting from 0.1 to 1.0 and then by evaluating the sensitivity of the method as defined by Eq. 2. Since in our model the objective of optimization is the sensitivity, we have forced unlikely fitness to configurations that are unable to filter out a reasonable number of SNPs. To control the filtering capability, we decide to ensure that the ratio between filtered SNPs and the total amount of SNPs considered must be lower than the threshold $T_{r}$, defined as:

(4) $$T_{r}=\frac{\left|Y\right|}{\left|S\right|}$$

**Figure 2:**
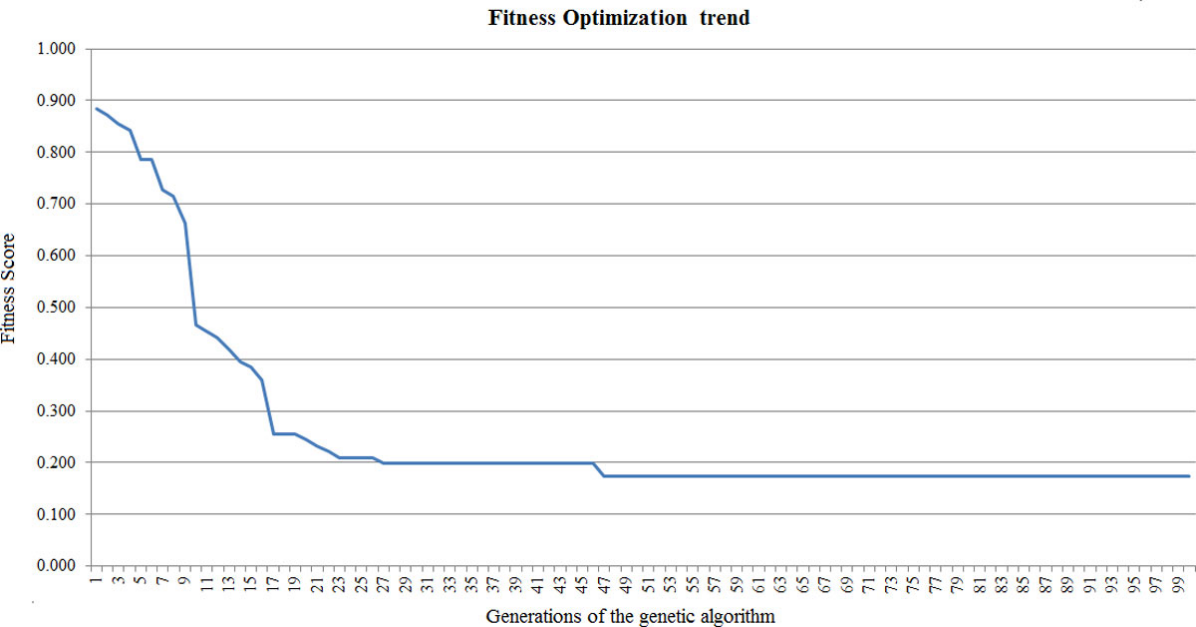
**Fitness optimization trend of the genetic algorithm**. The figure shows that the optimization algorithm is able to minimize the fitness value within the limit of 100 generations.

In order to estimate how well our predictive model performs, we chose to exploit the Leave One Out Cross Validation approach. This model implies that a single disease of the considered set is used as validation data, while the remaining pathologies are used for training the model. This operation is repeated for all the pathologies in the set used for this machine learning approach, such that each disease is employed once as validation data. Then, the average of the sensitivity in each test case is evaluated in order to compute the predictive capability. The genetic algorithm was run considering 100 generations, each of which composed by 120 individuals. The top 50 individuals are selected for the next generation. In detail, in each generation 10 new randomly generated individuals are added to the population, 10 individuals are taken unmodified from the previous generation and 100 individuals are generated by the recombination of the best 50 individuals of the previous generation. Figure 2 shows the fitness trend during the optimization.

In this way, a total of 640 simulations were run, by choosing iteratively one of the 16 pathologies as test set and evaluating 10 steps of $T_{\varepsilon }$ (from 0.1 to 1.0) and 4 steps of $T_{r}$ (from 0.25 to 1.0). Once all the simulations were completed, we validated the parameters configurations against each disease previously chosen as test case for such simulation: for each validation test, we evaluated the fitness with Eq. 2 and we determined the sensitivity, the specificity, and the accuracy of that parameter configuration. Then, we calculated the average values of such indexes for all the 16 simulations with the same $T_{\varepsilon }$ and $T_{r}$, in order to estimate how accurately our predictive model will perform in practice. All the data relative to genetic evolutions of parameter configurations have been collected according to same values of $T_{\varepsilon }$ and $T_{r}$, in order to estimate the performance of the predictive model, as reported in Additional File 3. The best parameters for assigning higher scores to diseases associated SNPs determined using our machine learning approach are visible as default feature weights in the SNPranker 2.0 home page. Pathologies, however, are characterized by different traits, and so each parameter configuration may work better with certain diseases rather than others. For this reason, users can directly set each feature weight on the basis of the aims of their specific study. This fine-tuning procedure is possible by associating each feature to a weight that represents the importance attributed by users to the feature in the final SNP score computation.

### Scoring computation through web interface

We developed the web site using object oriented programming languages exploiting PHP and JavaScript technologies. Performances are improved using the NDB engine of the MySQL Cluster, which works as backend database, in order to minimize times needed for query executions. Through the web interface, users can run analyses using three types of input: a list of genes, a biological process, and a list of SNPs. For each of these three choices, it is possible to perform the ontological expansion, customizing the similarity measure and its relative scoring threshold.

Once users complete their selections and start the computation, SNPranker 2.0 first extracts all the SNPs related to the selected genes (or biological process), then computes the ontological expansion (if required), and finally computes the score for all the input SNPs according to the selected features and weights. For each SNP, all selected features are presented in a final table combined with each score, showing both the original data, for annotation purpose, and the related scores. The web interface displays on the fly all the results and, at the end of the computation, output SNPs can be effectively ranked according to their scores, which are available in the last column of the output table. When the result page is completely loaded, a link to download output data in compressed format is presented at the bottom of the table and the SNP list enrichment tools about pathways and diseases become available to users.

### Dataset enrichment analysis

The enrichment of the ranked SNP list, considering KEGG pathways [46], GO [47] terms and OMIM gene and genetic disorders [31], is a valuable tool to interpret the output of the system. For example, considering the annotation of the top ranked SNPs in terms of KEGG pathways, it is possible to verify if the system has privileged genomic features belonging to a particular biological network. At the same way, an enrichment of best hits in a particular genetic disorder according to OMIM can be a clear indication that identified SNPs are effectively involved in a specific disease. The enrichment is computed by comparing the total number of genes that have a particular ontological annotation with respect to the number of top ranked genes with the same annotation (considering the genes that bring the identified SNPs). Statistical significance of the enrichments is assessed with appropriate hypergeometric tests, which permit to verify if the number of occurrences of a particular ontological annotation in the top ranked list of SNPs is by chance. Due to the high number of *P*-values computed for this analysis, the statistics is corrected using the False Discovery Rate control method [48], using the "phyper", "dhyper" and "p.adjust" routines available in R [49].

## Results

**Figure 3:**
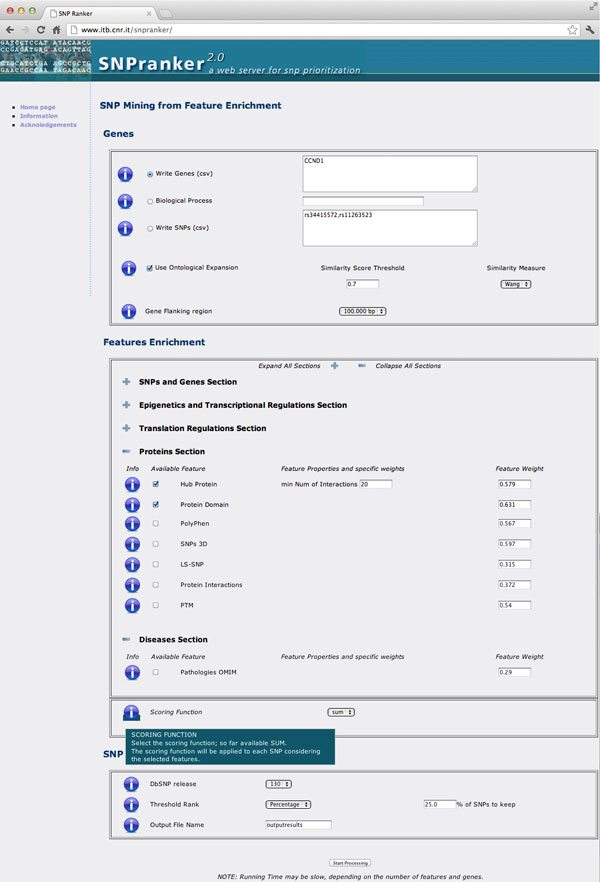
**SNPranker 2.0 home page screenshot**. A screenshot of the SNPranker 2.0 home page, with feature sections collapsed and expanded: the protein section gives an example of the available features with their relative weights. At the bottom, in the scoring function section, an information balloon is opened.

SNPranker 2.0 is available to users through a web interface accessible at the URL http://www.itb.cnr.it/snpranker. Figure 3 shows a screenshot of the home page.

### System input and features selection

The system takes as input a list of genes, a set of SNPs or a biological process. Genes and SNPs can be provided as comma separated values of IDs (EntrezGene or GeneSymbol for genes, RS identifiers for SNPs). For the biological process option, the web interface provides an auto-completion box with the GO names of biological processes. Once a particular biological process is selected, all genes annotated with this GO term are provided as input to the system. Since many SNPs are not directly associated to genes because of their inter-genic localization, SNPranker 2.0 provides a parameter for customizing the flanking regions.

### Ontological expansion

The ontological expansion is an important method for studying SNPs related to pathologies, since it allows to extend the analysis to SNPs that could potentially be involved in a pathology onset, but are not annotated as disease associated and have not being highlighted in more traditional approaches. The inclusion in the computation of SNPs belonging to genes that are annotated similarly to those provided as input permit to increase the number of associated SNPs under analysis. For this reason, the ontological expansion enriches the input list *g_1 _*by adding new genes that are biologically related with them, relying on GO terms. The biological relationship among genes is evaluated through two semantic similarity metrics (referred as Rel and Wang in the web page), which compare the GO terms associated with each gene. Depending on the interests of the user, for each gene in *g_1_*, the system retrieves a number of genes with the highest semantic similarity according to their Gene Ontology annotations.

### Features set

The home page of SNPranker 2.0 shows to users all the available features for the final SNP ranking, grouped by semantic and functional characterization. Default values of feature weights derive from our machine learning algorithm and are listed in Table 1. Although the huge presence of SNPs in introns, these are localized at lower positions within the ranked list obtained with the optimised feature weights, while at the top of it many "frame shift", "missense" and "start codon" are concentrated. Other SNPs occur, in order of appearance, hence of decreasing importance, in "start codon", "missense", 3' and 5' UTR regions, or even 3' and 5' near gene. Importantly, users can customize the set of features to process for the final score both by selecting or deselecting each feature and by modifying the weights of the features according to their expertise. Tuning a weight means changing the relevance of a biomolecular aspect, which reflects on the final SNP prioritization. For instance, if users are interested in studying the effect of a polymorphism on the abundance of a transcript, they can assign higher relevance to SNPs occurring in regulatory regions, such as the 5' near gene region.

**Table 1:** Default feature weights as result of the optimization process.

Section	Feature Name	Feature Weight
SNPs and Genes	MAF	0.3133
	Localization	0.7052
	Essential Genes	0.2665
	Phylo	0.5797
	Lamina associated domains	0.2444

Epigenetics and transcription regulations	Open Chromatin	0.1596
	Chromatin Structure	0.7525
	Methylation (seq regions)	0.4009
	Methylation	0.3743
	CpG Island	0.8992
	DNase clusters	0.9558
	TSS (eponine)	0.3705
	CpG islands, promoters, first exons	0.9665
	FOX2 CLIP-seq	0.5608
	TAF1 binding sites	0.2468
	Intergenic regulatory elements	0.4006
	TSS (SwitchGear)	0.6773
	Regulatory regions (OregAnno)	0.8818
	TFBS (TRANSFAC)	0.8243
	TXN factor ChIP-Seq	0.4477
	Enhancers (VISTA)	0.9571

Translation regulations	Alternative Splicing	0.7032
	miRNA binding regions	0.8358

Proteins	Hub protein	0.5796
	Protein Domain	0.6316
	PolyPhen	0.5678
	SNPs 3D	0.5977
	LS-SNP	0.3158
	Protein Interactions	0.3728
	PTM	0.5399

Disease	Pathologies OMIM	0.2904

The table presents the SNP features used by SNPranker 2.0 with their default weights, according to the optimization performed using the genetic algorithm (sensitivity = 0.814, specificity = 0.761 and accuracy = 0.761).

A description of each feature is available within the web interface in an intuitive balloon text close to each feature name. For example, considering the epigenetics features, a user can select a particular tissue or a cell line, while in case of selection of the general feature only, without the detail of tissue or cell line, the average values are considered for the score computation.

### SNP ranking

Once all values have been computed, the last step consists in ranking all SNPs relying on their scores. Due to the great amount of SNPs potentially reported as output, SNPranker 2.0 allows users to cut the list at a given threshold, based on a percentile of the total number of SNPs. Moreover, the enrichment tools allow testing if the provided SNP list is enriched of genes associated with a particular disease or pathway. The final list of enriched SNPs with scores is specifically aimed at supporting the evaluation of disease associated SNPs.

## Discussion

The SNPranker 2.0 tool has been validated using OMIM data, considering a set of pathologies influenced by recognized SNPs. For each disease the list of associated genes has been given as input to the system and the list of ranked SNPs has been compared to the set of SNPs provided by OMIM for the same disease.

Optimal feature weights were found in order to obtain the best sensitivity, which is the system capability of detecting correct cases, but other indexes such as specificity, which evaluate the capability of the system to filter incorrect cases, and accuracy, which measure the degree of closeness of our classification to real cases, should be taken into account. This is due to the fitness dependence on $T_{r}$ (the ratio between high scored SNPs and the total number of evaluated SNPs), which makes sensitivity more unfavourable in case of higher $T_{r}$.

Therefore, fitness values proposed by the machine learning algorithm should be carefully considered, because simulations that can identify almost all the disease associated SNPs do not filter out SNPs with the same effectiveness, and so the specificity and the accuracy indexes tend to lower values. Vice versa, greater values of accuracy and specificity mean less predictive power of the system.

The use of $T_{r}=0.25$ and $T_{\varepsilon }=0.3$ seems reasonable, since it results in 81% of associated SNPs with an accuracy and specificity of 76%. In some considered cases, (such as Cystic Fibrosis, Sickle Cell Anaemia, and Haemophilia) the top ranked SNPs show a statistically significant enrichment (*P *< 0.05, hypergeometric test) concerning SNPs known to be associated with the tested pathologies. In Huntington's disease, the first three SNPs appearing in the ranked list are exactly those reported in OMIM for this pathology.

We tested SNPranker 2.0 using different parameters and here we discuss two case studies: the first scenario is a search for semantic annotation and the second case is a comparison with a GWAS output.

In the first case we started from a gene, BCL2, known be associated with a disorder, B-Cell Cll/Lymphoma 2, as reported in OMIM. We collected all pathologies related to BCL2 from the Genecards database [50] and we looked at these disorders into OMIM, finding related genes. In Genecards, BLC2 is mentioned in different disorders, from lymphoma to cancer and leukaemia. We queried OMIM for the most important genes associated to these pathologies and we obtained a list of genes. We validated SNPranker 2.0 results considering output genes (those associated with the provided ranked SNPs) with respect to this list and we found that most disease related genes are effectively identified by an ontological expansion performed using the Wang similarity measure with threshold of 0.2. Table 2 (upper rows) reports these results, summarizing the similarity scores.

**Table 2:** Semantic similarity analysis of tested genes.

OMIM Disorder	Gene Symbol		Similarity score
		
	Input Gene	Known Associated	
B-Cell Cll/Lymphoma 2	BCL2	CDKN2A	0.389
		MYC	0.305
		TP53	0.434
		BRCA1	0.382
		BRCA2	0.329
		CCND1	0.247
		ATM	0.370

Bipolar disorder	KLF12	RORA	0.636
		RORB	0.759
		ARNTL	0.636
		HTR2A	0.301

Given two scenarios of different disorders, the table shows the similarities, computed using the Wang metrics score, among genes that are known to be associated with the pathologies and the ontologically enriched output gene lists.

For the second case, we compared our results with the tests of Chen et al. [51] looking at the "bipolar disorder" in the GWAS catalogue [52], using the reference of Le-Niculescu et al. [53]. Considering the gene *KLF12 *as input, since it is mentioned as one of the most important gene related to bipolar disorders, the ontological enrichment using the Wang similarity measure with a threshold of 0.3 returns a large set of 532 genes that belong to similar GO biological processes. Considering these genes, we confirmed that 52% of disease genes have been correctly returned, while the remaining are genes that could indirectly affect disease associated genes. Table 2 (lower rows) summarizes the similarity scores that we obtained from our ontological similarity enrichment given the input gene *KLF12*, with respect to known disease genes. The list of output prioritized SNPs, computed with default weights, returned most of the confirmed disease associated SNPs, according to Chen et. al [51] data, as reported in Table 3. We noted that scores slightly depends on user settings and thus outputs can differ among diverse input parameters set up. Statistically, the more genes are semantically similar to the input ones the more their related SNPs appear at higher positions within the ranked list.

**Table 3:** SNPranker results comparison with a GWAS for Bipolar Disorder.

Gene	SNP ID	Chr	Position		Strand	Alleles	Function
ARNTL	rs900145	11	13250480	13250481	-	A/G	unknown (intergenic)
HTR2A	rs1575891	13	47096716	47096717	+	C/T	unknown (intergenic)
KLF12	rs9543325	13	72814628	72814629	+	C/T	unknown (intergenic)
KLF12	rs1886512	13	73418186	73418187	+	A/T	intron
RORA	rs3743266	15	58568804	58568805	-	A/G	unknown (UTR-3)
RORA	rs340029	15	58682256	58682257	+	C/T	intron
RORA	rs3784609	15	58697841	58697842	-	A/G	intron
RORA	rs11071559	15	58857279	58857280	+	C/T	intron
RORA	rs12912233	15	59054387	59054388	+	C/T	intron
RORA	rs809736	15	59117079	59117080	+	A/G	intron

Given the best results of the GWAS concerning the Bipolar Disorder [53], the table shows the SNPs that have been correctly predicted in our final SNPs table.

## Conclusions

Given the need of tools for SNP prioritization, we updated our prototype system by developing SNPranker 2.0, a web based system that performs data mining of public available biomolecular knowledge of SNPs. SNPranker 2.0 is based on a gene-centric data-warehouse approach, which exploits a machine learning method to rank SNPs and compute final scores. It relies on the identification of a set of crucial features characterizing SNPs related to a list of input genes. This represents the *a priori *knowledge that employing our data mining approach allows the assessment of a final score for each SNP, which can be tuned by users according to their preferences. By employing a genetic algorithm we created a supervised classifier, which estimates the optimal weights of the SNP features. Using these parameters, SNPranker 2.0 provides a scored list of variations, which can be statistically analysed to verify its enrichment about particular pathways or diseases genes. Concrete scenarios of usage are the identification of the most important SNPs in population genetics studies, in order to create custom genotyping chips, and GWAS output re-scoring for interpreting top ranked SNPs in a specific biological context.

## Competing interests

The authors declare that they have no competing interests.

## Authors' contributions

IM conceived the study, developed the reference database, and drafted the manuscript. AC conceived of the study, developed the web interface, designed the scoring algorithm, and drafted the manuscript. PC implemented the machine learning algorithm, provided the results of the analysis and optimized system performances. FV developed the reference database, identified the test cases, and drafted the manuscript. EM participated in the design of the study, formalized the optimization problem, and worked at the enrichment analysis. LM coordinated the project, granted access to the computational facilities and maintained the bioinformatics resources. All authors read and approved the final manuscript.

## Declarations

The publication costs for this article were funded by the Italian Ministry of Education and Research (MIUR) through the Flagship (PB05) "InterOmics" project.

This article has been published as part of *BMC Bioinformatics *Volume 14 Supplement 1, 2013: Computational Intelligence in Bioinformatics and Biostatistics: new trends from the CIBB conference series. The full contents of the supplement are available online at http://www.biomedcentral.com/bmcbioinformatics/supplements/14/S1.

## Supplementary Material

Additional File 1 **SNPranker 2.0 features set**. All the available SNP features at SNPranker 2.0 web site, grouped in semantic sections. Click here for file

Additional file 2 **The OMIM diseases employed for the machine learning approach**. The table shows the list of diseases employed for training the scoring algorithm, providing information about the genomics regions, the disease names, the OMIM disease IDs, and the involved genes, summarized as gene symbols and Entrez IDs. Click here for file

Additional file 3 **Results of the genetic algorithm optimization process**. For each disease of the training set, the table summarizes SNP counts, sensitivity, specificity and accuracy achieved with the optimal feature weights found with the genetic algorithm. Click here for file
