# Supplementary material for: SNPranker 2.0: a gene-centric data mining tool for diseases associated SNP prioritization in GWAS
Source: BMC Bioinformatics. 2013 Jan 14;14(Suppl 1):S9. doi: 10.1186/1471-2105-14-S1-S9 (PMC3548692; doi:10.1186/1471-2105-14-S1-S9)
Supplement: Additional File 1 — SNPranker 2.0 features set. All the available SNP features at SNPranker 2.0 web site, grouped in semantic sections. [file 1471-2105-14-S1-S9-S1.PDF]

Additional File 1 - SNPranker 2.0 features set.

All the available SNP features at SNPranker 2.0 web site, grouped in semantic sections.

Section	Feature Name	Feature Description
SNPs and Genes	MAF	The Minor Allele Frequency represents the frequency of the less frequent allele of a SNP in a specific population
	Localization	This feature describes if a SNP is '5 near', '5 UTR', 'missense', 'nonsense', 'synonymous', 'frame shift', 'intron', '3 UTR' or '3 near'
	Essential Genes	It provides a score to SNPs which are within essential genes
	Phylo	Data about the phylogenetic derivation of the polymorphisms
	Lamina associated domains	It describes genome sites that interact with nuclear lamina components
Epigenetics and transcription regulation	Open Chromatin	It displays open chromatin regions and transcription factor binding sites accessibility in multiple cell types
	Chromatin Structure	Data about the chromatin structure and DNA/SNPs accessibility to transcription in multiple cell types
	Methylation (seq regions)	Sequencing data about methylation regions and transcription capabilities in genome for different cell types
	Methylation	Data about methylation of SNPs and subsequent Transcription Factor accessibility for multiple cell types
	CpG Island	It indicates if SNPs occur in genomic regions that contain a high frequency of CpG sites
	DNase clusters	It considers if a SNP is in a DNase hypersensitive area (promoters, enhancers, silencers, insulators, and locus control regions) for multiple cell types
	TSS (eponine)	It indicates Transcription Start Sites, estimated using the 'eponine' probabilistic method
	CpG islands, promoters, first exons (firstEF)	It considers predictions from the First Exon Finder program about exon, promoter and CpG window
	FOX2 CLIP-seq	It shows adaptor-trimmed CLIP-seq reads that map uniquely to the repeat-masked human genome

	TAF1 binding sites	It maintains the binding sites of the TATA box binding protein associated factor
	Intergenic regulatory elements	The data identify the intergenic regulatory elements
	TSS (SwitchGear)	It describes the location of Transcription Start Sites throughout the human genome along with a confidence measure based on experimental evidence
	Regulatory regions (OregAnno)	It presents literature curated regulatory regions, Transcription Factor binding sites, and regulatory polymorphisms from Open Regulatory Annotation
	TFBS (TRANSFAC)	It contains the location and score of Transcription Factor binding sites conserved in the human/mouse/rat alignment
	TXN factor ChIP-Seq	It shows regions where Transcription Factors bind to DNA as assayed by ChIP-seq
	Enhancers (VISTA)	It reports the distant-acting transcriptional enhancers in the human genome, as identified by the VISTA Enhancer Browser
Translation regulation	Alternative Splicing	Data about patterns able to regulate RNA alternative splicing
	miRNA binding regions	It considers if a SNP is involved in a miRNA binding region
Proteins	Hub protein	Evaluation of the number of protein-protein interactions established by a protein
	Protein Domain	It considers SNPs in the coding sequence that causes variations in the protein amino acid sequence
	PolyPhen	This feature provides information about predictions of non-synonymous SNPs effects on protein function
	SNPs 3D	Data about molecular functional effects of non-synonymous SNPs relying on structure and sequence analysis
	LS-SNP	It provides annotations about non-synonymous polymorphisms
	Protein Interactions	Database about the numbers and types of interactions for proteins
	PTM	Data about Post Translational Modification in proteins
Disease	Pathologies OMIM	It indicates if the considered SNP is identified as directly involved in a specific pathology
