# Supplementary material for: SNPranker 2.0: a gene-centric data mining tool for diseases associated SNP prioritization in GWAS
Source: BMC Bioinformatics. 2013 Jan 14;14(Suppl 1):S9. doi: 10.1186/1471-2105-14-S1-S9 (PMC3548692; doi:10.1186/1471-2105-14-S1-S9)
Supplement: Additional file 2 — The OMIM diseases employed for the machine learning approach. The table shows the list of diseases employed for training the scoring algorithm, providing information about the genomics regions, the disease names, the OMIM disease IDs, and the involved genes, summarized as gene symbols and Entrez IDs. [file 1471-2105-14-S1-S9-S2.PDF]

Additional File 2 - The OMIM diseases employed for the machine learning approach

The table shows the list of diseases employed for training the scoring algorithm, providing information about the genomics regions, the disease names, the OMIM disease IDs, and the involved genes, summarized as gene symbols and Entrez IDs.

Genomic Region	Disease Name	OMIM ID	Gene Symbol and ID	
4p16.3	Achondroplasia	100800	FGFR3	134934
12p13.31	Alzheimer disease, susceptibility to	104300	A2M	103950
17q23.3	Alzheimer disease, susceptibility to	104300	ACE	106180
7q36	Alzheimer disease-10	104300	AD10	609636
12p11.23-q13.12	Alzheimer disease-5	104300	AD5	602096
10q24	Alzheimer disease 6	104300	AD6	605526
20p	Alzheimer disease 8	104300	AD8	607116
19p13.2	Alzheimer disease 9, late onset	104300	AD9	608907
4p13	Alzheimer disease, late-onset	104300	APBB2	602710
21q21.2|21q21.3	Alzheimer disease 1, familial	104300	APP	104760
17q11.2	Alzheimer disease, susceptibility to	104300	BLMH	602403
6p21.3	Alzheimer disease, susceptibility to	104300	HFE	613609
17q23.1	Alzheimer disease, susceptibility to	104300	MPO	606989
7q36	Alzheimer disease, late-onset	104300	NOS3	163729
7q36	Alzheimer disease, susceptibility to	104300	PACIP1	608254
10q24	Alzheimer disease, late-onset	104300	PLAU	191840
11q23.2-q24.2	Alzheimer disease, pathogenesi	104300	SORL1	602005
2p13	Amyotrophic lateral sclerosis, susceptibility to	105400	DCTN1	601143
12q12-q13	Amyotrophic lateral sclerosis, susceptibility to	105400	PRPH	170710
21q22.1|21q22.11	Amyotrophic lateral sclerosis, susceptibility to	105400	SOD1	147450
22q12.2	Amyotrophic lateral sclerosis, susceptibility to	105400	NEFH	162230
15q11.2	Angelman syndrome	105830	UBE3A	601623
Xp22	Angelman syndrome-like	105830	CDKL5	300203
Xq28	Angelman syndrome	105830	MECP2	300005

11q22-q23	Ataxia-telangiectasia	208900	ATM	607585
14q32.32|14q32.32	Breast cancer, somatic	114480	AKT1	164730
11q22-q23	Breast cancer, susceptibility to	114480	ATM	607585
2q34-q35	Breast cancer, susceptibility to	114480	BARD1	601593
13q12.3	Breast cancer, susceptibility to	114480	BRCA2	600185
17q22.2	Breast cancer, early-onset	114480	BRIP1	605882
2q33-q34	Breast cancer, protection against	114480	CASP8	601763
16q22.1	Breast cancer, lobular	114480	CDH1	192090
22q11|22q12.1	Breast cancer, susceptibility to	114480	CHEK2	604373
5q33.2-qter	Breast cancer, susceptibility to	114480	HMMR	600936
12p12.1	Breast cancer, somatic	114480	KRAS	190070
6pter-q12	Breast cancer, susceptibility to	114480	NQO2	160998
16p12.2	Breast cancer, susceptibility to	114480	PALB2	610355
17q21	Breast cancer, susceptibility to	114480	PHB	176705
3q26.3	Breast cancer, somatic	114480	PIK3CA	171834
17q23.2	Breast cancer	114480	PPM1D	605100
15q15.1	Breast cancer, susceptibility to	114480	RAD51A	179617
1p32	Breast cancer, invasive ductal	114480	RAD54L	603615
8q11	Breast cancer, somatic	114480	RB1CC1	606837
11p15.5	Breast cancer, somatic	114480	SLC22A1L	602631
17p13.1	Breast cancer	114480	TP53	191170
14q32.3	Breast cancer, susceptibility to	114480	XRCC3	600675
7q31.2	Cystic fibrosis	219700	CFTR	602421
19q13.2|19q13.1	Cystic fibrosis lung disease, modifier of	219700	TGFB1	190180
Xp21.2	Duchenne muscular dystrophy	310200	DMD	300377
1q21	Gaucher disease, type I	230800	GBA	606463
Xq28	Hemophilia A	306700	F8	300841
17q11.2	Neurofibromatosis, type 1	162200	NF1	613113
1p36.21	Pancreatitis, chronic, susceptibility to	167800	CTRC	601405
5q32	Pancreatitis, hereditary	167800	SPINK1	167790
7q31.2	Pancreatitis, idiopathic	167800	CFTR	602421
7q32-qter|7q34	Pancreatitis, hereditary	167800	PRSS1	276000
12q22-q24.2	Phenylketonuria	261600	PAH	612349
9q31.1	Tangier disease	205400	ABCA1	600046
6q23.3	Refsum disease	266500	PEX7	601757
10p13	Refsum disease	266500	PHYH	602026
13q14.2	Retinoblastoma	180200	RB1	614041
11p15.5	Sickle cell anemia	603903	HBB	141900
6p23	Spinocerebellar ataxia 1	164400	ATXN1	601556
1p36.32	Zellweger syndrome	214100	PEX10	602859

1p36.22	Zellweger syndrome	214100	PEX14	601791
1q23.2	Zellweger syndrome	214100	PEX19	600279
2p16.1	Zellweger syndrome	214100	PEX13	601789
6q24.2	Zellweger syndrome, complementation group G	214100	PEX3	603164
7q21.2	Zellweger syndrome-1	214100	PEX1	602136
12p13.31	Zellweger syndrome	214100	PEX5	600414
22q11.21	Zellweger syndrome	214100	PEX26	608666
