# Supplementary material for: SNPranker 2.0: a gene-centric data mining tool for diseases associated SNP prioritization in GWAS
Source: BMC Bioinformatics. 2013 Jan 14;14(Suppl 1):S9. doi: 10.1186/1471-2105-14-S1-S9 (PMC3548692; doi:10.1186/1471-2105-14-S1-S9)
Supplement: Additional file 3 — Results of the genetic algorithm optimization process. For each disease of the training set, the table summarizes SNP counts, sensitivity, specificity and accuracy achieved with the optimal feature weights found with the genetic algorithm. [file 1471-2105-14-S1-S9-S3.PDF]

Additional File 3 - Results of the genetic algorithm optimization process

For each disease of the training set, the table summarizes SNP counts, sensitivity, specificity and accuracy achieved with the optimal feature weights found with the genetic algorithm.

**Disease Name**	**Total**	**Filtered**	**OMIM**	**True**	**False**	**True**	**False**	**Sensitivity**	**Specificity**	**Accuracy**	**Training**
Hemophilia A	1,275	253	20	20	233	1,022	0	1.000	0.814	0.817	0.209
Duchenne Musc Distrophy	11,789	516	2	1	515	11,272	1	0.500	0.956	0.956	0.165
Zellweger Syndrome	11,864	811	5	1	810	11,049	4	0.200	0.932	0.931	0.159
Retinoblastoma	2,251	1,326	1	1	1,325	925	0	1.000	0.411	0.411	0.174
Refsum Disease	3,350	190	5	3	187	3,158	2	0.600	0.944	0.944	0.171
Phenylketonuria	2,032	1,497	16	16	1,481	535	0	1.000	0.265	0.271	0.169
Alzheimer Disease	18,990	1,076	4	4	1,072	17,914	0	1.000	0.944	0.944	0.169
Cystic Fibrosis	3,352	1,432	4	4	1,428	1,920	0	1.000	0.573	0.574	0.169
Breast Cancer	30,629	3,413	11	8	3,405	27,213	3	0.727	0.889	0.889	0.184
SLA	4,969	282	4	1	281	4,684	3	0.250	0.943	0.943	0.325
Tangier Disease	2,756	194	3	3	191	2,562	0	1.000	0.931	0.931	0.190
Neurofibromatosis	2,951	251	1	1	250	2,700	0	1.000	0.915	0.915	0.163
Gaucher Disease I	1,121	284	4	3	281	836	1	0.750	0.748	0.748	0.169
AtaxiaTelangiectasia	2,195	1,462	3	3	1,459	733	0	1.000	0.334	0.335	0.202
Sickle Cell Anemia	2,593	570	2	2	568	2,023	0	1.000	0.781	0.781	0.176
Achondroplasia	1,160	243	2	2	241	917	0	1.000	0.792	0.792	0.165
Counts	103,277	13,800	87	73	13,727	89,463	14	-	-	-	-
**Mean**	**6,454.813**	**862.500**	**5.438**	**4.563**	**857.938**	**5,591.438**	**0.875**	**0.814**	**0.761**	**0.761**	**0.185**
**Std. Deviation**	**8,166.092**	**846.957**	**5.477**	**5.585**	**845.827**	**7,607.800**	**1.360**	**0.282**	**0.235**	**0.234**	**0.040**
Max	30,629	3,413	20	20	3,405	27,213	4	1.000	0.956	0.956	0.325
Min	1,121	190	1	1	187	535	0	0.200	0.265	0.271	0.159
